# Inbreeding depression in red deer calves

**DOI:** 10.1186/1471-2148-11-318

**Published:** 2011-10-31

**Authors:** Craig A Walling, Daniel H Nussey, Alison Morris, Tim H Clutton-Brock, Loeske EB Kruuk, Josephine M Pemberton

**Affiliations:** 1 Institute of Evolutionary Biology, School of Biological Sciences, University of Edinburgh, Edinburgh, EH9 3JT, UK; 2 Centre for Infection, Immunity and Evolution, School of Biological Sciences, University of Edinburgh, Edinburgh, EH9 3JT, UK; 3 Department of Zoology, University of Cambridge, Cambridge, CB2 3EJ, UK

## Abstract

**Background:**

Understanding the fitness consequences of inbreeding is of major importance for evolutionary and conservation biology. However, there are few studies using pedigree-based estimates of inbreeding or investigating the influence of environment and age variation on inbreeding depression in natural populations. Here we investigated the consequences of variation in inbreeding coefficient for three juvenile traits, birth date, birth weight and first year survival, in a wild population of red deer, considering both calf and mother's inbreeding coefficient. We also tested whether inbreeding depression varied with environmental conditions and maternal age.

**Results:**

We detected non-zero inbreeding coefficients for 22% of individuals with both parents and at least one grandparent known (increasing to 42% if the dataset was restricted to those with four known grandparents). Inbreeding depression was evident for birth weight and first year survival but not for birth date: the first year survival of offspring with an inbreeding coefficient of 0.25 was reduced by 77% compared to offspring with an inbreeding coefficient of zero. However, it was independent of measures of environmental variation and maternal age. The effect of inbreeding on birth weight appeared to be driven by highly inbred individuals (F = 0.25). On the other hand first year survival showed strong inbreeding depression that was not solely driven by individuals with the highest inbreeding coefficients, corresponding to an estimate of 4.35 lethal equivalents.

**Conclusions:**

These results represent a rare demonstration of inbreeding depression using pedigree-based estimates in a wild mammal population and highlight the potential strength of effects on key components of fitness.

## Background

Inbreeding depression, the reduction in fitness of offspring resulting from matings between related individuals, is of considerable importance in studies of evolution, ecology and conservation [for reviews see [1-4]]. However, although there are now many studies demonstrating the existence of inbreeding depression in laboratory and captive populations, there are still relatively few examples in natural populations [4]. Such studies are important because inbreeding may affect extinction risk in small populations of conservation interest [5] and because patterns of inbreeding and inbreeding depression seen in laboratory and captive populations may not be representative of those seen in natural populations [3,6,7]. For example, laboratory and captive populations experience relatively stable and benign environments, but there is increasing evidence that inbreeding depression may vary with environmental conditions [reviewed in [8,9]]. In addition, recent studies have highlighted the potential for interactions between inbreeding depression and age-related variation in fitness traits [e.g. [10,11]] but we know relatively little about the impact that age-related variation may have on the realisation of inbreeding depression in natural populations [though see [12]].

One reason for the lack of studies of inbreeding depression in natural populations is the difficulty in collecting the data required to estimate levels of inbreeding. An individual's inbreeding coefficient F, defined as the probability that two alleles at any randomly-chosen locus are identical by descent [13], can be calculated from pedigree records, but doing so accurately requires multiple generations of pedigree data, which may not always be available. Thus, as a proxy for pedigree-based inbreeding coefficients, a number of studies of natural populations have used measures of multilocus heterozygosity (MLH) from variable markers such as allozymes and microsatellites, on the assumption that MLH will decline linearly with increasing F [reviewed in [14-16]]. However, recent studies have demonstrated that the correlation between measures of heterozygosity at small numbers of marker loci and pedigree-based calculations of inbreeding coefficients are typically weak [17,18], questioning the validity of the assumption that MLH accurately captures variation in inbreeding coefficient [19,20]. Here we used pedigree-based inbreeding coefficients to investigate the effect of inbreeding on the juvenile traits birth date, birth weight and first year survival in a wild population of red deer (*Cervus elaphus*) on the Isle of Rum, Scotland.

Interest in the potential for environmental conditions and age to affect the magnitude of inbreeding depression, either in life history components or in morphometric traits has a long history in laboratory populations [8,21-23]. For example, numerous studies of *Drosophila *have demonstrated that the magnitude of inbreeding depression is dependent on the environment experienced [e.g. [9,23-27]] and this is also a well known effect in plants [28-30]. In general, the pattern appears to be one of increasing inbreeding depression in more stressful environments [reviewed in [8,22]]. In terms of the association between inbreeding depression and age, attention has focussed on whether or not inbreeding depression increases with age as a result of the reduction in the strength of selection with age predicted by evolutionary theories of ageing [31,32] and evidence for an increase in inbreeding depression with age is growing for laboratory studies [10,11,31,33-35].

However, studies of the association between inbreeding depression and either environmental severity or age in natural populations are rare. One notable exception is the population of song sparrows (*Melospiza melodia*) found on Mandarte Island, Canada [36]. Here, there is evidence for an increase in inbreeding depression in more severe environments [37,38], but mixed support for an increase in inbreeding depression with age [12]. Examples in other species include increasing inbreeding depression in more severe environments in wild populations of great tits (*Parus major*) [7] and of cactus finches (*Geospiza scandens*) [39], but Kruuk et al. [40] found no evidence for such an interaction in a population of collared flycatchers (*Ficedula albicollis*). Age-related variation in inbreeding is less well studied in natural populations: in addition to the results of Keller et al. [[12] - above] Wilson et al. [41] found evidence for an increase in inbreeding depression on a measure of individual annual fitness [pti - [42]] with age in the same population of red deer as used in the current study, although the traits contributing to this association were not investigated.

The majority of studies of inbreeding depression have concentrated on effects on traits expressed in early life such as juvenile survival or growth rate [2], which can be considered as both a trait of the individual and of the mother [43]. In animals with extended periods of maternal care, such as birds and mammals, maternal age and/or condition has a substantial impact on such traits. For example, numerous offspring traits show an initial increase with maternal age, thought to be associated with changes in maternal experience or condition, followed by a plateau at prime age and a subsequent decline in older age as maternal performance senesces [e.g. [44-46]]. Despite this strong effect, we know relatively little about the relationship between maternal age and inbreeding depression on juvenile traits. Given the theory and observations presented above, maternal age could be predicted to influence inbreeding depression in juvenile traits in two ways. First, treating juvenile traits as traits of the mother, if the strength of selection declines with increasing age and thus inbreeding depression increases with age, we might predict that maternal inbreeding coefficient should interact with age such that there is an increase in inbreeding depression on juvenile traits in inbred mothers at older ages. However, treating juvenile traits as traits of the offspring, if maternal age indicates the quality of the maternal environment experienced by offspring, with young and old mothers providing relative poor environments, then we might also predict an interaction between offspring inbreeding coefficient and maternal age, with inbreeding depression being more severe for inbred offspring born to young and old mothers than for inbred offspring born to prime aged mothers. To the best of our knowledge these hypotheses have yet to be tested.

**Figure 1:**
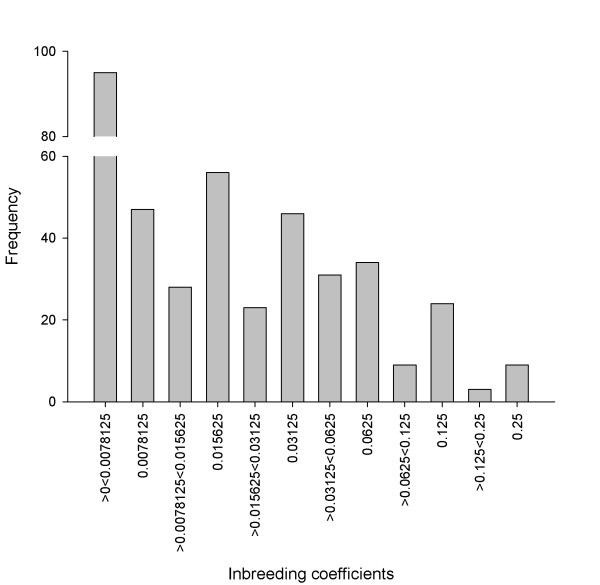
**The distribution of inbreeding coefficients (F) for individuals with F>0**. Inbreeding events are split as in table 1. Although there is evidence for some reasonably close inbreeding events (father-daughter (F = 0.25), half-siblings (F = 0.125)) a number of individuals have inbreeding coefficients resulting from mating between more distantly related individuals (F>0<0.0078125).

Here we investigate the effects of inbreeding on the juvenile traits birth date, birth weight and first year survival in a wild population of red deer (*Cervus elaphus*) on the Isle of Rum, Scotland. Previous studies of this population have revealed positive associations between marker-based measures of inbreeding (multilocus heterozygosity and mean d^2 ^[47]) and birth weight [47,48], neonatal survival [47,48], first winter survival [49] and lifetime reproductive success [50], but at the time of the previous work sample sizes for pedigree-based inbreeding coefficients were too small for analysis [47]. Since then, continued data collection and improvements in the pedigree [51] have increased the number of individuals with pedigree-based inbreeding coefficients of greater than zero (Figure 1), allowing a pedigree-based approach to studying inbreeding depression. Further, effects of maternal age and environmental variables have been demonstrated in this population for a number of juvenile traits, including those studied here [44,49,52-56]. Therefore in this study, we use pedigree-based inbreeding coefficients to investigate inbreeding depression at both the maternal and offspring level in key early-life traits, and to investigate the relationship between environmental variation, maternal age and inbreeding depression. We also calculate the number of lethal equivalents [[57] see methods for details] for first year and first winter survival as a standardized measure of inbreeding depression to allow comparison with other studies [e.g. [4,58]].

## Results

### Levels of inbreeding

Considering individuals born between 1980 and 2010 for which both parents and at least one grandparent were known, 405/1848 (21.9%) had a pedigree based inbreeding coefficient (F) of greater than 0 (Figure 1), with a mean F of 0.00724. Restricting the dataset to individuals with all four grandparents known this increased to 346/821 (42.1%), with a mean F of 0.0128, suggesting the frequency of inbreeding is underestimated as a result of incomplete pedigree information. However, within inbred individuals the proportion of different inbreeding events was relatively similar between the two datasets (table 1). Close inbreeding events (F = 0.25) made up 2.2% of non-zero inbreeding events and resulted exclusively from father-daughter matings, probably as a result of the mating system in this species and the rarity of full sibs [59]. Although a reasonable proportion of inbred individuals are a result of relatively close inbreeding (19.5% of inbred individuals have *F*≥0.0625; i.e. a mating between first cousins or closer relatives), a number of inbred individuals also have low but non-zero levels of inbreeding (23.5% of individuals have 0>*F*<0.0078125; i.e. a less than half-second cousin mating) and the majority of inbred individuals have moderately low levels of inbreeding (57.0% of inbred individuals have 0.00778125≥*F*<0.0625; i.e. between half-first cousin or second cousin mating). Thus the depth of pedigree information available for this population allows us to detect numerous low level inbreeding events. Contrary to results from a highly inbred island populations of song sparrows [60] there was no correlation between maternal and offspring inbreeding coefficient (linear mixed effect model with mother as a random effect, estimate = 0.00451 ± 0.0568, F_1,390 _= 0.006, p = 0.94)

**Table 1:** Variation in the frequency of inbreeding events depending on the depth of the pedigree.

Inbreeding group	N (both parents and at least one grandparent known)	% of inbred	N (all 4 grandparents known)	% of inbred
>0.25	0	0.0	0	0.0
0.25	9	2.2	2	0.6
<0.25>0.125	3	0.7	3	0.9
0.125	24	5.9	23	6.6
<0.125>0.0625	9	2.2	8	2.3
0.0625	34	8.4	28	8.1
<0.0625>0.03125	31	7.7	27	7.8
0.03125	46	11.4	40	11.6
<0.03125>0.015625	23	5.7	21	6.1
0.015625	56	13.8	45	13.0
<0.015625<0.0078125	28	6.9	26	7.5
0.0078125	47	11.6	43	12.4
<0.0078125>0	95	23.5	80	23.1
Total with F>0	405	100	346	100
Total with F = 0	1443		475	

Inbreeding events are split into well known groups (e.g. 0.25 - parent-offspring, full-siblings etc; 0.125 - half-siblings, uncle-niece etc; 0.0625 - first cousin, half-uncle-niece etc; 0.03125 - first cousins once removed, half-first cousins etc; 0.015625 - second cousins, first cousins twice removed etc; 0.0078125 - second cousins once removed, half-second cousins etc.) and those that require more complex relationships. The relative frequency of types of inbreeding event is similar when considering datasets of individuals with both parents and at least one grandparent known or with all four grandparents known.

### Offspring birth date

Birth date varied with birth year, maternal status and a quadratic function of maternal age (table 2). Birth dates were earlier for individuals born to prime-aged mothers than for those born to young or old mothers (table 2). However, there was no evidence of an effect of either maternal or offspring inbreeding coefficient on offspring birth date (table 2). There was also no significant interaction between either maternal or offspring inbreeding coefficient and the fixed effects of maternal age, its quadratic, autumn rainfall, population size, sex or the random effect of year of birth (at removal all interactions p > 0.1).

**Table 2:** Minimal generalised linear mixed models of birth date and offspring birth weight.

	Birth date (N = 2515 calves, 602 mothers)			Birth weight (N = 1664 calves, 487 mothers)		
**Random effects **	**Variance ± SE**	**p**		**Variance ± SE**	**p**	

Mother ID	26.29 ± 4.67	<0.001		0.626 ± 0.231	<0.001	
Year of birth (YOB)	8.46 ± 2.58	<0.001		0.0461 ± 0.0170	<0.001	
YOB*Offspring F	*0*^*B*^	*1*		*2.04 ± 8.15*	*0.775*	
YOB*Mother's F	*0*^*B*^	*1*		*0*^*B*^	*1*	
Residual	267.8 ± 8.53	<0.001		0.707 ± 0.260	<0.001	

**Fixed effects**	**Estimate ± SE**	**Wald F_df_**	**p**	**Estimate ± SE**	**Wald F_df_**	**p**

Mother's age	-2.55 ± 0.67	14.53_1,2489_	<0.001	0.394 ± 0.050	61.50_1,1454_	<0.001
Mother's age^2^	0.154 ± 0.033	21.39_1,2493_	<0.001	-0.0224 ± 0.0025	77.48_1,1440_	<0.001
Mother's status^a ^TY	-7.35 ± 0.94	25.56_4,2472_	<0.001	0.616 ± 0.067	34.11_4,1439_	<0.001
N	-5.30 ± 1.47			0.0236 ± 0.104		
SY	-10.12 ± 1.19			0.559 ± 0.087		
WY	-1.42 ± 1.34			-0.157 ± 0.086		
Birth weight	NF			NF		
Birth date	NF			0.0131 ± 0.0021	39.08_1,1564_	<0.001
Population size	-0.139 ± 0.035	15.76_1,28_	<0.001	*0.00277 ± 0.00286*	*0.94*_*1,31*_	*0.340*
Sex^b^	*0.301 ± 0.765*	*0.15*_*1,1719*_	*0.689*	0.358 ± 0.047	59.27_1,1405_	<0.001
Environmental variable	0.0171 ± 0.0074^c^	5.35_1,27_	0.029	0.181 ± 0.065^d^	7.79_1,33_	0.009
Offspring F	*3.26 ± 13.7*	*0.06*_*1,1345*_	*0.805*	-2.325 ± 0.983	5.6_1,1507_	0.019
Mother's F	*42.5 ± 30.5*	*1.95*_*1,318*_	*0.166*	*3.33 ± 3.28*	*1.03*_*1,405*_	*0.312*

NF term not fitted in model. ^a^Reference for mother's status is milk (M) hinds. ^b^Reference level for sex is females. ^c^Autumn rainfall, ^d^Spring temperature. ^B^Bound at zero. Final models were achieved by sequentially dropping the least significant terms based on Wald statistics until only significant terms remained. Denominator degrees of freedom are calculated numerically using a Kenward and Roger adjustment in ASReml [88]. Text in italics indicates non-significant parameters and their associated estimates and significance at removal (see methods for details). N = sample sizes for minimal models.

### Offspring birth weight

**Figure 2:**
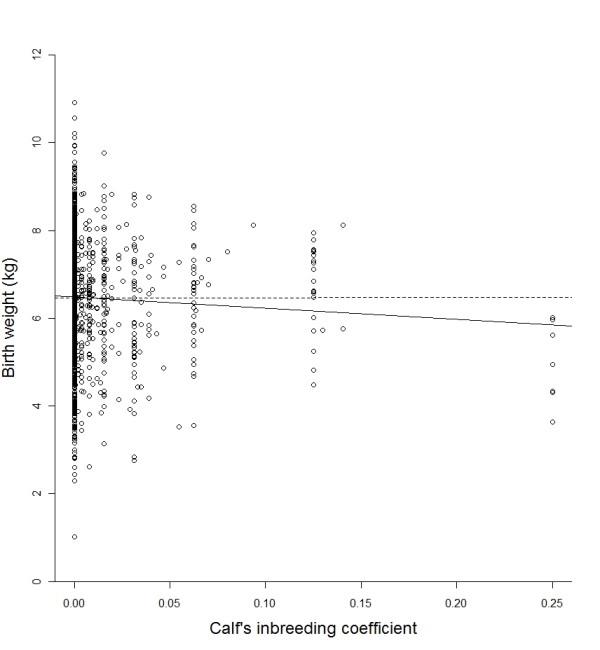
**The effect of offspring inbreeding coefficient on birth weight in red deer**. Solid line represents the least squares regression line (i.e. not correcting for other terms in the mixed model) including all inbreeding coefficients, dashed line represents the least squares regression line with offspring with inbreeding coefficients of 0.25 removed. Calves with higher inbreeding coefficients are born lighter, but this seems to be a result of the reduced birth weight of highly inbred (F = 0.25, father-daughter matings) calves.

Offspring inbreeding coefficient had a significant effect on offspring birth weight, with more inbred offspring being lighter at birth (table 2, Figure 2). Birth weight was also affected by the sex of the calf, the mother's status, the mother's age as a quadratic term, birth date and average spring temperature (table 2). However, there was no evidence for a significant effect of the mother's inbreeding coefficient (table 2) nor for any significant interactions between offspring or maternal inbreeding coefficient and any of the environmental or age variables fitted (at removal, all p > 0.14). The effect of offspring inbreeding coefficient appeared to be driven by the low birth weight of highly inbred calves (F = 0.25, Figure 2); removal of these 7 individuals (2 calves with F = 0.25 had no birth weight record) rendered the effect of offspring inbreeding coefficient on birth weight non-significant (coefficient=-1.301 ± 1.231, F_1,1373 _= 1.12, p = 0.293, Figure 2).

### First year survival

**Figure 3:**
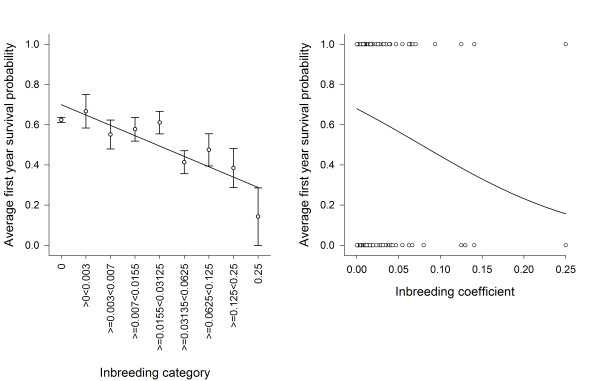
**The association between an offspring's inbreeding coefficient and its probability of first year survival**. The left-hand plot shows averages of raw data (so not correcting for terms in the minimal model) for offspring with inbreeding coefficients binned as detailed on the x-axis, with bars representing standard errors. The right hand plot shows model predictions from a model including birth weights (solid line) and actual data (open circles; please note that a given circle may represent multiple data points, e.g. for F = 0.25, there was 1 survivor and 6 non-survivors). Offspring were less likely to survive their first year with increasing inbreeding coefficient.

The probability of calves surviving the first year of life decreased significantly with increasing inbreeding coefficient (table 3, Figure 3). In line with previous research [61], we found a strong and significant effect of birth weight on first year survival (table 3), but individual's inbreeding coefficient clearly also influenced first year survival independent of birth weight. Indeed removal of birth weight from the model only changed the parameter estimate of the effect of offspring inbreeding coefficient from -9.74 ± 2.29 to -8.68 ± 2.47. There was no significant effect of maternal inbreeding coefficient (table 3) nor any significant interaction between offspring or maternal inbreeding coefficient and any of the environmental or age related variables modelled (at removal, all p > 0.07). Maternal status, maternal age and its quadratic were all non-significant in these models (table 3), although it should be noted that they were significant in models of first winter survival (table 3, below). Limiting this analysis to individuals with inbreeding coefficients less than 0.25, the effect of offspring inbreeding coefficient remained significant (coefficient=-8.56 ± 3.21, χ^2^_1 _= 7.11, p = 0.008). Similarly, limiting the dataset to only individual with all four grandparents known, the effect of inbreeding coefficient remained significant (coefficients=-7.33 ± 3.32, χ^2^_1 _= 4.88, p = 0.027). Applying both restrictions the effect became marginally non-significant (coefficients=-6.65 ± 3.49, χ^2^_1 _= 3.62, p = 0.057)

**Table 3:** Minimal generalised linear mixed effects model for first year and first winter survival.

	First year survival (N = 1593calves, 463 mothers)			First winter survival (N = 1400 calves, 443 mothers)		
**Random effects**	**Variance ± SE**	**p**		**Variance ± SE**	**p**	

Mother ID	0.395 ± 0.126	<0.001		0.598 ± 0.184	<0.001	
Year of Birth (YOB)	0.705 ± 0.232	0.001		1.37 ± 0.44	<0.001	
YOB*Offspring F	*0*^*B*^			*0*^*B*^		
YOB*Mother's F	*354 ± 331*	*0.142*		*122 ± 350*	*0.364*	
Residual	1^fixed^			1^fixed^		

**Fixed effects**	**Estimate ± SE**	**Wald χ^2^_df_**	**p**	**Estimate ± SE**	**Wald χ^2^_df_**	**p**

Mother's age	*-0.0351 ± 0.0190*	*3.43*_*1*_	*0.064*	-0.0898 ± 0.0284	9.99_1_	0.002
Mother's age^2^	*0.00540 ± 0.00665*	*0.66*_*1*_	*0.42*	*-0.00391 ± 0.00787*	*0.25*_*1*_	*0.619*
Mother's status^a ^TY	*0.280*	*8.54*_*4*_	*0.074*	-0.301	10.5_4_	0.033
N	*0.468*			-0.512		
SY	*-0.196*			-0.0632		
WY	*-0.580*			-0.780		
Birth weight	0.580 ± 0.057	105_1_	<0.001	0.648 ± 0.074	76.5_1_	<0.001
Birth date	-0.0305 ± 0.0051	36.6_1_	<0.001	-0.0357 ± 0.0065	30.1_1_	<0.001
Population size	-0.0189 ± 0.0091	4.28_1_	0.039	-0.0257 ± 0.0126	4.14_1_	0.042
Sex^b^	-0.528 ± 0.125	17.7_1_	<0.001	-0.741 ± 0.153	23.4_1_	<0.001
Environmental variable	-0.0044 ± 0.0020^c^	4.5_1_	0.034	-0.0069 ± 0.0029^c^	5.59_1_	0.018
Offspring F	-9.74 ± 2.29	11.3_1_	<0.001	-15.2 ± 3.3	19.6_1_	<0.001
Mother's F	*0.509 ± 5.46*	*0.01*_*1*_	*0.926*	*4.07 ± 6.81*	*0.36*_*1*_	*0.550*

^a^Reference for mother's status is milk (M) hinds. ^b^Reference level for sex is females. ^c^Winter rainfall. ^fixed^Residual variance is fixed at zero because it is not estimable for binary data. ^B^Bound at zero. Final models were achieved by sequentially dropping the least significant terms based on Wald statistics until only significant terms remained. Text in italics indicates non-significant parameters and their associated estimates and significance at removal (see methods for details). N = sample sizes for minimal models.

Decomposing first year survival into summer and winter survival indicated that the effect of offspring inbreeding coefficient on first year survival is driven by its effect on winter survival (coefficient=-15.2 ± 3.43, χ^2^_1 _= 19.6, p < 0.001, table 3). There was no effect of offspring inbreeding coefficient (at removal, coefficient = 3.56 ± 3.86, χ^2^_1 _= 0.85, p = 0.356) or maternal inbreeding coefficient (at removal, coefficient = 6.47 ± 5.20, χ^2^_1 _= 1.55, p = 0.213) on summer survival; the only parameters to have a significant influence on summer survival were birth weight (coefficient = 0.351 ± 0.057, χ^2^_1 _= 37.9, p < 0.001) and birth date (coefficient=-0.0142 ± 0.0049, χ^2^_1 _= 8.33, p = 0.004). For first winter survival, limiting the dataset to only individuals with all four grandparents know, the effect of offspring inbreeding coefficient remained significant (coefficient=-10.8 ± 3.79, χ^2^_1 _= 8.07, p = 0.004), as it did when removing highly inbred (F = 0.25) individuals (coefficient=-14.8 ± 3.57, χ^2^_1 _= 17.2, p < 0.001). Applying both restrictions gave identical results to restricting to individuals with four grandparents known, because no individuals with F = 0.25 and four grandparents known survived the summer.

### Number of lethal equivalents

The number of lethal equivalents was estimated for first year survival and winter survival as these were the survival traits that showed clear evidence of inbreeding depression (see above). For first year survival, there was statistical support for variation in the survival of non-inbred individuals between years (${Ŝ}_{0,y}$ ranged from 0.16 (1992) to 1 (1980), χ^2^_29 _= 254.97, p < 0.001). The maximum likelihood estimate of *B *from the model where ${Ŝ}_{0,y}$ varied was 4.35 (95% CI = 2.15-7.06). However there was no support for variation in the number of lethal equivalents (*B*) between years (χ^2^_29 _= 32.87, p = 0.282). The same pattern was true for winter survival, with support for variation in ${Ŝ}_{0,y}$ between years (${Ŝ}_{0,y}$ ranged from 0.21 (1992) to 1.0 (1980), χ^2^_29 _= 305.33, p < 0.001) but not for variation in *B *between years (χ^2^_29 _= 33.71, p = 0.250). The maximum likelihood estimate of *B *for winter survival from the model where ${Ŝ}_{0,y}$ varied was 4.75 (95% CI = 2.65-7.36).

## Discussion

The results of this study represent a rare examination of the effects of inbreeding in a wild mammal population using pedigree-based inbreeding coefficients as opposed to marker-based estimates of inbreeding [see also [62]]. We found evidence for inbreeding depression in offspring birth weight and offspring first year survival but not in birth date. Effects were driven by offspring inbreeding coefficients whereas maternal inbreeding coefficients had no significant effect on any of the juvenile traits studied. Inbreeding depression in birth weight appeared to be a result of the low birth weight of highly inbred individuals (F = 0.25) whereas inbreeding depression in first year survival was evident across all inbreeding levels. The effect of inbreeding on first year survival was quite severe, with the average survival probability of individuals with F of 0.25 being less than 0.15 compared to 0.62 for outbred individuals (F = 0, Figure 3) and the number of lethal equivalents estimated as 4.35. This was driven by effects on first winter survival rather than summer survival. We found no statistical support for any interactions between the effects of inbreeding and our measures of environmental variables, sex or age.

### Comparison with marker-based results

Previous work on this population has focussed on marker-based estimators as a proxy for inbreeding because sample sizes for pedigree based measures were previously too small [47]. In general, the patterns we found here are similar to the previous work, showing inbreeding depression for birth weight and offspring survival, but they differ in some details. Birth weight decreased with increasing values of the marker-based measures of inbreeding in both previous studies on this population [47,48], although here we found that this result was entirely dependent on the inclusion of the 7 individuals with F = 0.25. However, although there was no effect of inbreeding on summer survival in the current analysis, a previous analysis found that summer survival decreased with decreasing mean d^2^, although this effect was non-significant when accounting for birth weight [47]. Removing birth weight from the current analysis of summer survival, the effect of offspring inbreeding coefficient was still non-significant (effect = 0.231 ± 3.07, χ^2^_1 _= 0.01, p = 0.94). Furthermore, a previous analysis of winter survival found that the relationship was sex-specific when using mean d^2 ^calculated from up to nine microsatellite loci, being positive for females and negative for males [49], but non-significant using a larger panel of microsatellites and either mean d^2 ^or multilocus heterozygosity as measures [up to [71] loci, [48]]. In the current study, there was strong inbreeding depression in both first year survival and winter survival and no evidence for an interaction between sex and the effect of inbreeding (first year survival, χ^2^_1 _= 0.01, p = 0.934; winter survival, χ^2^_1 _= 0.17, p = 0.684). Reasons for these differences are unclear. They could represent differences in power: the current study has a larger sample size than any of the previous studies; between 2,515 and 1,592 observations, depending on the trait, in the present study versus 644 [47], 573 [49] and 364 [48] in previous studies, but this would not explain significant results in the previous studies but not in the current one. The differences could also be explained by the failure of marker-based measures to accurately reflect inbreeding, perhaps because they are a result of direct or local effects [63] [but see [20]].

### Interactions between inbreeding and environmental and age variation

The results of our analysis did not provide support for any interaction between the effect of inbreeding and environmental or age related variation in this population. Although rarely studied in natural populations, the general pattern of results from experimental populations and those natural populations where data are available is one of an increase in the detrimental effects of inbreeding with increasing environmental stress [e.g. [7,26,37-39,64] ] [for a review see [8]], although this is by no means ubiquitous [see for example [38,40,64]]. Previous analysis of this population suggested an interaction between average spring temperature and inbreeding effects on birth weight using a marker-based measures [mean d2 [47]] but there was no evidence for this using our pedigree-based measure (F_1,1108 _= 0.73, p = 0.392). Given that inbreeding depression in birth weight was driven by individuals with the highest inbreeding coefficients, it may be that the effects of such high levels of inbreeding are independent of environment. Inbreeding effects on offspring survival also showed no sign of interactions with environmental variation. The effect of inbreeding on offspring survival was strong and therefore perhaps independent of environmental variation, or it is possible that winter conditions are in general stressful enough that effects of inbreeding are independent of environmental variation. It is also possible that we lack the power to detect such effects, as suggested by the large standard errors on some of the fixed effects (e.g. effect of mothers inbreeding coefficient on birth date, table 2). For example, given that inbreeding depression on birth weight is driven by individuals with F = 0.25 and there are only 7 such individuals distributed across 6 unique years, we probably lack statistical power to determine the interaction effects of close inbreeding with environmental heterogeneity. However, overall sample sizes here are at least equivalent to other studies in natural populations where interactions have been demonstrated [38,39].

The relationship between age and inbreeding depression has been of considerable recent interest in laboratory studies, with tests concentrating on the prediction that inbreeding depression should increase with age as a result of weakening selection against deleterious alleles with increasing age [reviewed in [31,32]]. However, the results presented here suggest a general lack of an effect of maternal inbreeding coefficient on juvenile traits and thus no scope for any variation in this effect with maternal age. This may reflect an issue of statistical power: sample sizes for tests of maternal inbreeding coefficient were on average 73% smaller than those of offspring inbreeding coefficient (comparing number of unique mothers to number of unique offspring). However, if there is truly no effect of maternal inbreeding coefficient, this suggests that such traits in this system do not represent a good place to test for effects of age on inbreeding depression. Studying adult traits that show inbreeding depression that can therefore vary with age would be an interesting avenue of future research.

### Variation between traits

Patterns of inbreeding depression differed between the juvenile traits examined here. All effects of inbreeding were due to the offspring inbreeding coefficient, but birth date showed no clear evidence for inbreeding depression. Given that this trait is most likely determined by oestrus date and female condition during gestation [with gestation length showing relatively little variation in this population [65,66]], this may be a result of birth date being more a trait of the mother than of the offspring and thus offspring inbreeding coefficient having little effect. However, it should be noted that birth weight is also strongly influenced by maternal effects [see table 2, [54,67,68]]. Previously, Nussey et al. [44] have demonstrated that all of the juvenile traits studied here show age-dependent changes consistent with maternal senescence. The results of the current analysis suggest that most of these age-related changes in first year survival result from changes in birth weight. Inclusion of birth weight in the model of first year survival here resulted in the removal of the quadratic effect of female age; when birth weight was removed from the model, the quadratic effect of maternal age was significant (χ^2^_1 _= 7.41, p = 0.006). The same pattern was true for first winter survival with the quadratic effect of maternal age being marginally significant when birth weight was removed (χ^2^_1 _= 3.85, p = 0.050).

Although offspring birth weight is an important predictor of offspring first year survival, we found that offspring first year survival suffered strong inbreeding depression independent of the effects of inbreeding on birth weight. The first year survival of offspring with an inbreeding coefficient of 0.25 was reduced by 77% compared to offspring with an inbreeding coefficient of zero (Figure 3). The estimate of the number of lethal equivalents for first year survival was 4.35, which is relatively high compared to the range of estimates from natural populations of birds [39,40] and higher than the median estimate of 3.2 from captive mammal populations [58,69]. Estimates of lethal equivalents from natural mammal populations are rare, but although lower, our estimate was within the 95% confidence interval of two studies on natural populations of wolves (lethal equivalents were estimated as 6.05 (95% CI = 2.61-9.49) in Mexican wolves (*Canis lupus baileyi*) [70] and 5.19 (95% CI = 1.95-8.44) in a Swedish population of grey wolves (*Canis lupus*) [71]. Given that this effect appeared to be driven by inbreeding effects on winter survival and was independent of inbreeding effects on birth weight in models of survival including birth weight as a covariate, some later acting effects of inbreeding, for example on growth rate or resistance to winter stress over and above those due to birth weight, must be acting to reduce the probability of first winter survival for inbred offspring.

### The effect of incomplete pedigrees

Although the population of red deer studied here has one of the most complete pedigrees of any wild mammal population currently studied, approximately 40% of calves born to the study population have unknown paternity. Consequently the frequency of inbreeding estimated in this study is likely to be an underestimate (see results, levels of inbreeding) as is the severity of inbreeding depression [39]. Restricting our analyses to only individuals with all four grandparents known, all significant results presented here remained. However, the lack of evidence for any interactions between age or environment and inbreeding may be a result of the reduction in power caused by an incomplete pedigree. Although the correlation between heterozygosity estimated at a small number of loci (such as typical of microsatellite studies) and pedigree based estimates of inbreeding is poor [16-18], recent advances in the availability of high density molecular markers, such as single nucleotide polymorphisms (SNPs), are opening up the potential to calculate accurate inbreeding coefficients for individuals without pedigrees, with incomplete pedigrees and even founder individuals [72-74]. If these tools become available for this population, it will be interesting to see whether the improved power of these studies allows the detection of more subtle effects such as interactions with environment or age.

## Conclusion

This study presents an investigation of inbreeding effects in a wild mammal population using pedigree-based inbreeding coefficients as opposed to marker-based estimates. Although the results are generally consistent with previous studies using marker-based estimates in this population, differences do exist, highlighting the importance of confirming such associations with a pedigree-based approach. The fact that strong inbreeding depression for first winter survival was still apparent after controlling for birth weight highlights the potential for inbreeding to affect multiple facets of early-life performance in natural populations. Long-term studies of pedigreed natural populations such as this represent an ideal situation in which to generate a fuller understanding of inbreeding depression and particularly its context dependence, or lack of, under ecologically relevant conditions.

## Methods

### Study population

The red deer population resident in the North Block of the Isle of Rum, Inner Hebrides, Scotland (57° 03' N, 06° 21' W) has been the subject of intensive study since 1972 [53]. Individuals are recognisable from artificial tags or natural markings and are monitored through regular censuses throughout the year. During the winter, detailed mortality searches are carried out to locate carcasses of missing animals, giving accurate information on death date for the majority of individuals resident to the study area. Daily censuses are also carried out during the calving season (May to July) to accurately determine the date of birth for offspring of resident individuals. Approximately 80% of calves are caught soon after birth, weighed, artificially marked, and (since 1982) an ear punch taken for genetic analysis. This work is conducted under Project Licence # PPL60/3547 under the UK Animals (Scientific Procedures) Act 1986. Of individuals not caught at birth, some are sampled later either *post mortem *or by immobilisation. All sampled individuals are genotyped at up to 15 microsatellite loci [51,75,76].

### Inbreeding coefficients

Pedigree reconstruction was achieved using a combination of genetic and behavioural information as detailed in Walling et al. [51]. Briefly, maternity was known with certainty from behavioural associations between mothers and calves [77]. Paternity was assigned using a combination of genetic information and behavioural data in the parentage inference programs MasterBayes [78] and COLONY2 [79]. MasterBayes allows the simultaneous use of genetic and behavioural information relating to the likelihood of paternity, allowing more powerful paternity assignment. The behavioural information used here was a male's age and its square [80] and the length of time (days) that a male was seen holding a female in his harem around her estimated oestrus date [77]. Where MasterBayes failed to assign a sire at the requisite 80% individual confidence or higher, we used COLONY2 to assign individuals to paternal half-sibships. If the same ungenotyped male (not all individuals are genotyped in this population (see above)) was seen holding more than 50% of mothers of a particular paternal half-sibship around their estimated oestrus date, this male was assigned as the sire of all individuals within that paternal half-sibship; otherwise a dummy sire for the sibship was assigned. Further details of this procedure are given in Walling et al. [51].

Wright's inbreeding coefficient (F) was calculated for mothers and offspring from the pedigree using the software package PEDIGREE VIEWER version 6.4 http://www-personal.une.edu.au/~bkinghor/pedigree.htm[81,82]. F was only assigned to individuals with both parents and at least one grandparent known. Over 99% of detectable inbreeding events occurred after 1980, presumably because of lack of pedigree depth in earlier years, so we restricted analyses to data on individuals born between 1980 and 2010. Over this period, the dataset contained 3,121 calves, of which 2,919 had known maternity and 1,857 had known paternity; we therefore had estimates of F in 1,848 individuals born to 512 unique mothers, of whom estimates of F were available for 392.

### Measures used

We examined the effects of both an offspring's inbreeding coefficient and that of its mother on the juvenile traits birth date, birth weight and first year survival among all offspring caught at birth. Date of birth was scored as the number of days since 1st May in the year of birth, birth weight as capture weight (kg) minus 0.01539 times age at capture (hrs) [53]. First year survival was defined as 0 if the offspring was known to have died before 1st May of the calendar year following its birth year (i.e. in the first year of life), or 1 if it survived this period. We also further divided first year survival into summer survival (defined as survival from birth until 1st October of the year of birth) and winter survival (defined as survival from 1st October in the year of birth to 1st May of the subsequent calendar year) [53]. Thus having tested for inbreeding depression in first year survival, we subsequently ran models for summer and winter survival to assess which period was driving any effect. Individuals that were shot in culls outside our study area or that emigrated from the study population during their first year, and thus whose survival was unknown, were included in the analysis of birth date or birth weight but removed from the survival analyses. This gave inbreeding coefficients for a total of 1,834 individuals with known birth date of which 1,667 had known birth weights, 1,593 had known first year survival, 1,612 had known summer survival and 1,400 of which survived the summer and had known winter survival (193 individuals scored 0 for summer survival). Sample sizes for maternal inbreeding coefficients were 435 unique mothers for birth date, 407 for birth weight, 381 for first year survival, 388 for summer survival and 365 for winter survival (16 mothers had all offspring die during their first summer). Because fixed effects in the final models varied between traits, sample sizes for final models varied and are given in the results (tables 2 and 3).

In addition to the inbreeding coefficient of the offspring and its mother, a number of other fixed effects were fitted that are known to be important from previous studies. Offspring sex was fitted as a two-level factor in all models, as this is known to be an important predictor of birth weight and first winter survival, with males being heavier at birth but yet less likely to survive [53,55,83]. Previous analyses have found that birth date influences birth weight [55] and that both birth date and birth weight influence first year survival [61], thus these terms were fitted to the appropriate models. Environmental variables were selected on the basis of results from previous studies. These were: for birth date, total autumn rainfall (mm) between September and December in the autumn of gestation [55]; for birth weight, the average spring temperature (°C) between February and April [55]; and for first year survival, total winter rainfall (mm) between November and January of the first winter of life [49,54]. Environmental measures were taken from recordings on the island of Tiree, approximately 70 km south west of the study area [84]. Although climate data exists for Rum, it is incomplete and the correlation between temperature on Rum and Tiree is strong (r^2 ^> 0.94) [84]. Population size, measured as the number of adult females seen in ≥10% of censuses between January and May of the calendar year after birth, was also fitted to all models as it has been shown to have a significant effect on both birth date and first year survival [49,55]. Finally, the reproductive status of a mother and her age as a quadratic effect are important predictors of juvenile traits in this population [44,85,86]. In all models, reproductive status was fitted as a five-level factor describing the mother's recent reproductive history: Naive (N), female had not bred previously; True yeld (TY), female did not breed in the previous year; Summer yeld (SY), female bred in the previous year but the calf died before 1 October; Winter yeld (WY), female bred in the previous year but the calf died between 1 October and 1 May; Milk (M), the female successfully reared a calf in the previous year [85]. Maternal age in years was fitted as a quadratic function [44,85,86].

### Estimating the number of lethal equivalents

The number of lethal equivalents is a standardised measure of inbreeding depression in a population, defined as a gene or group of genes that would cause death in an individual in a homozygous state [57]. Assuming loci have independent effects, survival is expected to decline according to the equation: *S *= *S*_0_*e*^-*Bf *^where *S *is survival, *S_0 _*is the survival of non-inbred individuals, *B *is the number of lethal equivalents per gamete and F is the inbreeding coefficient under consideration. The parameter *B *is usually estimated as the regression coefficient from a least-squares linear regression of the natural log of survival against inbreeding coefficient. However, this method runs into problems when the average survival observed in a class of individuals with a particular value of F is zero. Kalinowski & Hedrick [87] suggest a maximum likelihood method of estimating *B *which avoids the zero-survival problem and Kruuk et al. [40] extended this to allow tests of between-year variation in both the survival of non-inbred individuals and in *B *[see [40] for details]. In brief, assuming a binomial model of survival, maximum likelihood estimates $\hat{B}$ and ${Ŝ}_{0}$ (and 95% confidence intervals) are estimated by maximising the log-likelihood function

(1) lnL= ∑iNsurv,i lnŜi+(Ntotal,i-Nsurv,i)ln(1-Ŝi),

where *N_surv,i _*is the number of survivors in class *i*, *N_total,i _*is the total number of calves in class *i*, ${Ŝ}_{i}={Ŝ}_{0}e^{-\hat{B}F_{i}}$ is the survival of individuals with inbreeding coefficient *F_i _*and the log-likelihood is summed over the different inbreeding classes *i*. The effect of allowing annual variation in the base-line survival ${Ŝ}_{0}$ is then assessed by maximising the log-likelihood function

(2) lnL= ∑y∑iNsurv,i lnŜy,i+(Ntotal,i-Nsurv,i)ln(1-Ŝy,i)

Where ${Ŝ}_{y,i}={Ŝ}_{0,y}e^{-\hat{B}F_{i}}$and *y *= 1980, 1981...2009. The significance of the effect of estimating ${Ŝ}_{0,y}$ rather than ${Ŝ}_{0}$ was assessed as -2 times the difference in the log-likelihoods between equations (1) and (2) on 29 d.f. (one minus the number of new parameters estimated (the number of years)). Adding between-year differences in *B *gives ${Ŝ}_{y,i}={Ŝ}_{0,y}e^{-{\hat{B}}_{y}F_{i}}$. Here, the difference in log-likelihoods from equation (2) and this model was compared on 29 d.f. (again one minus the number of new parameters estimated). All maximisation procedures were done in MICROSOFT EXCEL 2003 using the SOLVER tool as in Kalinowski & Hedrick [87] and Kruuk et al. [40].

### Statistical analyses

Birth date and birth weight are normally distributed and thus analysed using mixed models with normal errors using ASReml version 3.0 [88] to implement standard linear mixed effects models (i.e. not including the additive relationship matrix as a random effect). First year survival is a binary trait and was thus analysed in a generalised linear mixed model with binomial errors and a logit link function using Genstat Thirteenth Edition. All models contained the random effects of mother's identity and birth year, plus the fixed effects described above. These random effects account for the fact that the same mother contributed more than one offspring to the dataset and control for between-year variation not accounted for by fixed effects. To test for environmental and age dependence of inbreeding depression in juvenile traits, full models contained the interactions between offspring inbreeding coefficient and relevant fixed effects in the model: environmental variables (e.g. autumn rain for birth date), population size, mother's age, mother's age squared and sex. We also fitted a further random term of the interaction term between inbreeding coefficients and year of birth to test for year-to-year variation in inbreeding depression. The same interactions were also fitted with mother's inbreeding coefficient. Models were compared using a model simplification approach based on the removal of the least significant terms, using Wald statistics, until only significant terms remained. Identical results were obtained for the models of birth date and birth weight comparing models based on AIC scores (data not shown). The significance of random effects was assessed by comparing log-likelihoods of models with and without the terms fitted, with twice the difference in log-likelihood assumed to be Chi-square distributed with the number of degrees of freedom equal to the difference in the number of terms fitted to the models. Generalised linear mixed models use quasi-likelihoods rather than likelihoods and so log-likelihood values were not available for binary models. Significance for random effects in these models was therefore estimated on the basis of z-scores calculated as the ratio of the random effect estimate to the standard error on the estimate. Non-significant random effects were removed from minimal models. Minimal models were re-run following the removal of individuals with inbreeding coefficients of 0.25 to check if effects were being driven by individuals with high levels of inbreeding. In general the effects of inbreeding are assumed to be linear [81] and so we present the results of models under this assumption. In support of this, quadratic effects of inbreeding coefficient were not significant in any models (P ≥ 0.08).

In addition, the minimum inbreeding coefficient that can be assigned to an individual depends on the depth of the pedigree for that individual and this varies between individuals in this population (for the entire population (i.e. not restricted to those that F was calculated for), median = 3, minimum = 0, maximum = 9). To assess the consequences of this variation for our results we re-ran minimal models on a dataset restricted to individuals with all four grandparents known (sample sizes: Birth date 816 (344 with F>0), birth weight 749 (318 with F>0), first year survival 708 (296 with F>0), summer survival 716 (297 with F>0), winter survival 632 (266 with F>0)).

## Authors' contributions

CAW and DHN designed and carried out the statistical analysis. JMP, LEBK and THCB participated in the design of the analysis and are involved in the maintenance and management of the long-term monitoring project on Rum and JMP has led the sampling and genotyping of individuals. AM collected much of the data on which the analysis is based. CAW wrote the initial manuscript and DHN, JMP, LEBK and THCB all provided detailed comments and suggestions. All authors read and approved the final manuscript
